# Essentials of Medical Chemistry

**Published:** 1900-11

**Authors:** 


### Books and Magazines.

Essentials of Medical Chemistry, Organic and Inorganic, containing also Questions of Medical Physics, Chemical Philosophy, Anylitical Processes, Toxicology, Etc. Prepared especially for students of medicine by Lawrence Wolff, M. D., Demonstrator of Chemistry, Jefferson Medical College; Physician to the German Hospital of Philadelphia; Member of the Germal Chemical Society of the Philadelphia College of Pharmacy, Etc. Fifth edition. Thoroughly revised by Smith Ely Jelliffe, M. D., Ph. D., Professor of Pharmacology, College of Pharmacy of the City of New York, Etc. Philadelphia: W. B. Saunders, Publisher, 925 Walnut Street. 1899.

This little book contains 223 pages and sells for $1.00. The subject matter is arranged in the form of questions and answers, which allows a great deal to be put in a small space. The work is specially suited to students and to those who desire to run over the whole subject in a short time.

